# GA_3_ and Other Signal Regulators (MeJA and IAA) Improve Xanthumin Biosynthesis in Different Manners in *Xanthium strumarium* L.

**DOI:** 10.3390/molecules190912898

**Published:** 2014-08-25

**Authors:** Changfu Li, Fangfang Chen, Yansheng Zhang

**Affiliations:** CAS Key Laboratory of Plant Germplasm Enhancement and Specialty Agriculture, Wuhan Botanical Garden, Chinese Academy of Sciences, Wuhan 430074, China

**Keywords:** *Xanthium strumarium* L., glandular trichomes, xanthanolide, phytohormones

## Abstract

Xanthanolides from *Xanthium strumarium* L. exhibit various pharmacological activities and these compounds are mainly produced in the glandular trichomes of aerial plant parts. The regulation of xanthanolide biosynthesis has never been reported in the literature. In this study, the effects of phytohormonal stimulation on xanthumin (a xanthanolide compound) biosynthesis, glandular trichomes and germacrene A synthase (GAS) gene expression in *X. strumarium* L. young leaves were investigated. The exogenous applications of methyl jasmonate (MeJA), indole-3-acetic acid (IAA), and gibberrellin A_3_ (GA_3_) at appropriate concentrations were all found to improve xanthumin biosynthesis, but in different ways. It was suggested that a higher gland density stimulated by MeJA (400 µM) or IAA (200 µM) treatment caused at least in part an improvement in xanthumin production, whereas GA_3_ (10 µM) led to an improvement by up-regulating xanthumin biosynthetic genes within gland cells, not by forming more glandular trichomes. Compared to the plants before the flowering stage, plants that had initiated flowering showed enhanced xanthumin biosynthesis, but no higher gland density, an effect was similar to that caused by exogenous GA_3_ treatment.

## 1. Introduction

*X.* *strumarium* L. is an annual herb and its aerial parts have been used as a folk medicine to treat cancer, fever, headaches, leucoderma and skin pruritus [1]. The pharmacological activities are believed to be attributable to the presence of xanthanolide sesquiterpenes [2,3,4], that mainly occur in the glandular cells of the plant [5]. Using LC-MS and NMR, we recently identified three chemotypes of *X. strumarium* L. glandular trichomes, which are distributed from northern to southern areas of China. Among the three chemotypes, type I *X. strumarium* L. is the major species and is widely distributed within China [5]. Interestingly, *X. strumarium* L*.* is not the only plant species to produce xanthanolides, as these compounds are also produced in other species including *Helianthus annuus* [6,7] and *Carpesium longifolium* [8], indicating that the biochemical basis of xanthanolide biosynthesis may also be applicable to those species. The xanthanolide biosynthetic pathway is not clear, but these sesquiterpene compounds have been proposed to be derived from germacrene A [9]. The gene coding for germacrene A synthase (GAS) has been isolated from several plant species [10,11,12].

Despite the successful efforts towards the chemical synthesis of xanthanolide [13], to date the plant-based extraction remains the only actual source of the compounds for the market, therefore, it is important to evaluate the factors affecting xanthanolide production in *X. strumarium* L*.* However, the regulation of xanthanolide biosynthesis in *X. strumarium* L*.* has never been reported in the literature. The biosynthesis of plant secondary metabolites is usually developmentally and environmentally regulated [14]. Flowering is an important plant development event affecting sesquiterpene biosynthesis. For instance, the highest concentration of artemisinin (a sesquiterpene lactone with anti-malarial activity) has been detected at the flowering stage in *Artemisia annua* L. plants [15]. We speculated that the variation of innate hormone signals associated with flowering events might be the real driver for this appearance. For example, gibberellins (especially GA_3_) are the key molecules that promote flowering development [16], and also have been found to increase the formation of glandular trichomes [17] and sesquiterpene biosynthesis [14] in *Arabidopsis thaliana* plants. Therefore, in this study, we examined the effects of flowering initiation and exogenous GA_3_ on the formation of glandular trichomes and the biosynthesis of xanthumin on *X. strumarium* L*.* young leaves. In addition to GA_3_, other regulators such as methyl jasmonate (MeJA) [18] and indole-3-acetic acid (IAA) [19] are also reported to enhance isoprenoid production in plant cells, consequently the three phytohormones GA_3_, MeJA, and IAA were assessed for their effects on gland formation, xanthumin biosynthesis, and GAS gene expression in *X. strumarium*.

## 2. Results and Discussion

### 2.1. The Discrepancy between Gland Formation and Xanthumin Production upon Flowering Initiation

Flowering development has been shown to positively influence gland formation and sesquiterpene production in *A. annua* L., which tempted us to investigate whether similar effects could be adapted to the *X. strumarium* L*.* species. To induce flowering, a light treatment (12 h-light/12 h-dark cycles) was applied to 80 days-old seedlings. After 20–21 days, all the plants (100 days-old seedlings) started to flower. The first fully-expanded leaves from the top (a new born leaves) of the plants were collected and cut into two halves (see the Experimental Section) for analyzing xanthumin concentration and gland density, respectively. As shown in Table 1, relative to that of 80 days-old seedlings, neither gland density nor gland size was increased at the flowering initiation stage (showed by 100 days-old seedlings), whereas the concentration of xanthumin was improved by 36%. The observation indicated that the increase of xanthumin production by the flowering initiation was not correlated with the gland density. GA_3_ is an important molecule that plays overlapping roles in plant flowering development and secondary metabolism [15]. In this study, a higher endogenous GA_3_ level was also observed upon flowering initiation (shown by 100 days-old plants), which was 1.8-fold that of 80 days-old plants (Table 1). We hypothesized that the increased GA_3_ levels at the flowering stage might be the real factor responsible for the improved xanthumin biosynthesis, and to test this hypothesis, the effect of GA_3_ stimulus on xanthumin biosynthesis and gland formation in *X. strumarium* L*.* was investigated as described below.

**Table 1:** The effect of flowering initiation on the gland formation, xanthumin production and endogenous GA_3_ biosynthesis in young *X. strumarium* L. leaves. Bars are standard errors. Asterisks indicate significant differences. * *p* < 0.05, ** *p* < 0.01.

	Groups	Mean	SD	df	t	Sig. (p)
Trichome density (No./mm^2^)	80 days	4254.90	90.15	2		
	100 days	4029.82	315.16	2	−1.7325	>0.05
Cross-sectional area (µm^2^)	80 days	2305.15	140.75	2		
	100 days	2166.85	158.53	2	−13.4710	>0.05
Relative xanthumin level	80 days	1.00	0.13	2		
	100 days	1.36	0.01	2	−9.2520	<0.01 **
GA_3_ concentration (ng/g fresh weight)	80 days	0.37	0.04	2		
	100 days	0.69	0.08	2	13.6749	<0.05 *

For comparing the production of xanthumin, we set the average HPLC peak area of xanthumin of 80 days-old seedlings as 1, and that of 100 days-old seedlings was normalized thereto.

### 2.2. Effect of Phytohormonal Stimuli on Glandular Trichome Density and Xanthumin Production

**Figure 1:**
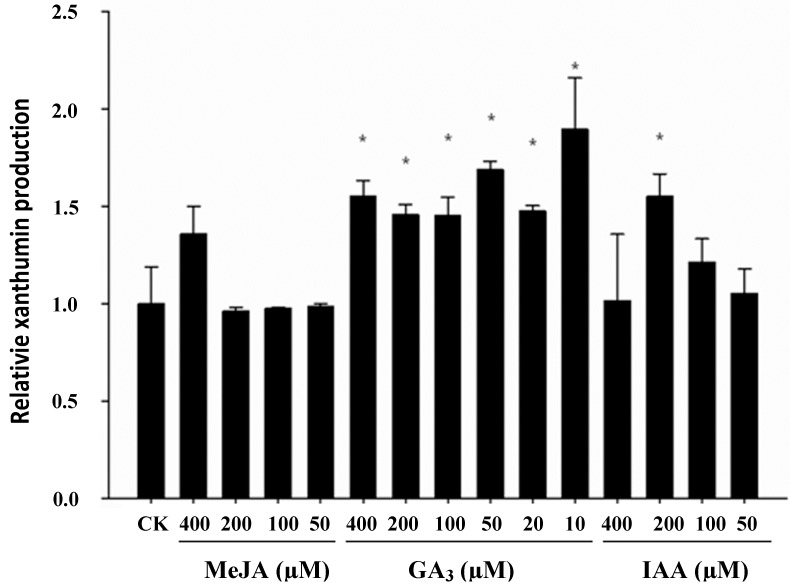
The effect of phytohormonal treatments on xanthumin accumulation in *X. strumarium* L*.* young leaves. We set the average HPLC area of xanthumin of the control plants as 1, and that of the hormones-treated plants was normalized thereto. Bars are standard errors. Asterisks indicate significant differences. * *p* < 0.05.

**Figure 2:**
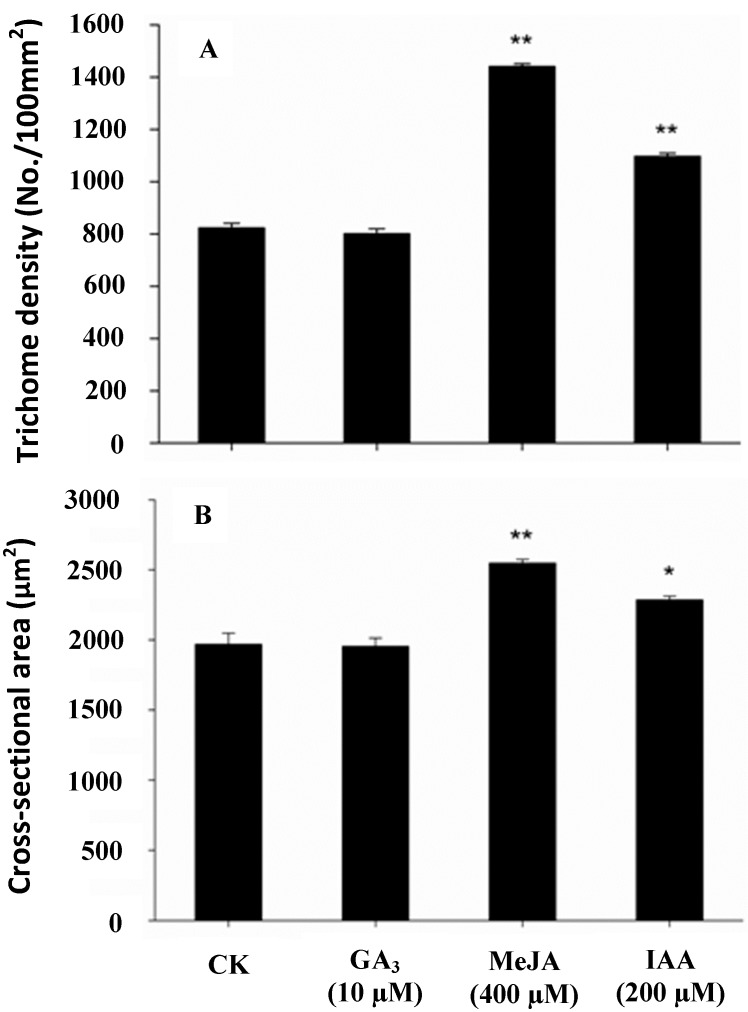
The effects of phytohormonal treatments on the density and size of glandular trichomes on *X. strumarium* L*.* young leaves. Bars are standard errors. Asterisks indicate significant differences. * *p* < 0. 05, **p < 0.01.

To test whether gland density and xanthumin production was affected in GA_3_-elicited *X. strumarium* L*.* plants, 25 days-old seedlings were treated with varying concentrations of GA_3_. In comparison with the control plants, the GA_3_-treated plants showed a longer internodal stem, which is a common phenotype in response to GA_3_ elicitation, while there were no obvious differences in leaf size. As shown in Figure 1, relative to the controls, a marked increase in xanthumin accumulation was stimulated by GA_3_ at all the concentrations, the lower concentration (10 µM) seemed to be more effective in triggering xanthumin biosynthesis, showing a 90% increase as compared with the control plants. Between the treatments with 20–400 µM of GA_3_, the increment trend of xanthumin concentration was not dose-dependent, indicating a certain degree of saturation for the elicitation with a higher concentration of GA_3_ (Figure 1). Meanwhile, the GA_3_ stimulus effects on gland density and gland size were examined as well. As shown in Figure 2, GA_3_ did not stimulate more gland formation or increase the size of glandular trichomes. A similar GA_3_ effect on gland formation was previously observed with *A. annual* L. plants. In that study, only 6-benzylaminopurine (BAP) and jasmonic acid (JA), but not GA_3_, stimulated glandular cell formation on *A. annual* L. leaves [20]. In contrast to the effects on Asteraece plants, GA_3_ treatment could promote trichome proliferation in *A. thaliana* leaves [17]. The mechanisms underlying the epidermal differentiations might be different in the model plant *A. thaliana* and the Asteraece plant family. Taken together, these observations indicated that GA_3_ did not stimulate more xanthumin production by causing a higher gland density. The results would also explain for the discrepancy between the gland formation and xanthumin production upon flowering initiation described above (see Figure 1 and Figure 2).

To test whether the GA_3_ effect is unique or applicable to other plant hormones, another two plant hormones, MeJA and IAA, were assessed for their effects on xanthumin production and gland formation in *X. strumarium*. In comparision with the control plants, no visible inhibitions on leaf growth were observed with both treatments. As shown in Figure 1, for the MeJA treatment, only 400 µM was able to increase xanthumin production and no improvements were observed at the other concentrations. For the IAA elicitation, IAA triggered an increase of xanthumin accumulation in a dose-dependent manner in the 50–200 µM concentration range, suggesting a positive effect on xanthumin production. Relative to the controls, 100 µM IAA only slightly increased xanthumin production, while the elicitation by 200 µM IAA increased the xanthumin level in a significant manner. The optimal concentrations of MeJA (400 µM) and IAA (200 µM) were then chosen for monitoring their effects on gland cells. As shown in Figure 2, MeJA or IAA treatment increased both the gland density and gland size relative to the mock controls, which was not observed with the GA_3_ treatment described above. Considering that the three phytohormones all increased xanthumin biosynthesis but exerted different effects on gland formation, we demonstrated that they stimulated more of xanthumin production by employing different strategies. For MeJA or IAA applications, the formation of more of glandular trichomes, resulted, at least in part, in a higher level of xanthumin production, whereas GA_3_ improved xanthumin biosynthesis not by forming higher gland density, but possibly by up-regulating xanthumin biosynthetic genes.

### 2.3. The Increased Expression of Germacrene A Synthase Gene Induced by GA_3_ Treatment

**Figure 3:**
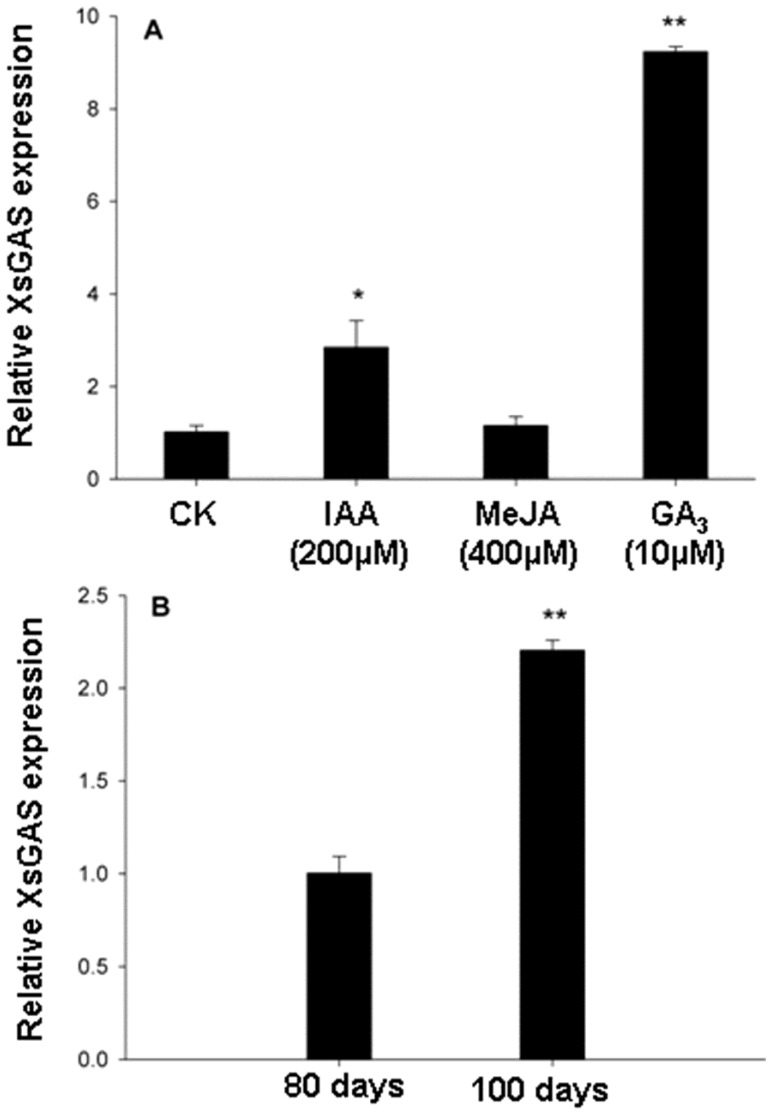
The effects of flowering initiation and phytohormonal treatments on *XsGAS* expression in *X. strumarium* L. young leaves. Data presented are representative of two independent experiments. Error bars indicate the range of possible value on SD of replicate 2^−ΔΔC^_T_ values. Asterisks indicate sisgnificant differences. * *p* < 0.05, ** *p* < 0.01.

To start understand how GA_3_ modulates xanthumin production, we examined the GAS transcript variation upon the phytohormonal treatments. Xanthumin belongs to the xanthanolides which have been suggested to be derived from germacrene A [9]. A glandular trichome specifically-expressed GAS cDNA (GenBank accession no. KJ194511), named *XsGAS*, has been cloned from *X. strumarium* L*.* by our group and was functionally confirmed by expression in *E. coli* and *in vitro* enzyme assays (unpublished data). XsGAS displays 83%–90% amino acid identities with several previously identified germacrene A synthases from other plant species (Figure S1). The variations of *XsGAS* transcript level in response to GA_3_ treatment were evaluated by qRT-PCRs. The PCR efficiency of *XsGAS* and the reference actin gene is about 40% and 50%, respectively, judging by the difference in the fluorescence strength between PCR cycles. As shown in Figure 3A, relative to the control, GA_3_ and IAA treatments increased the transcript level of *XsGAS* by 8.1-fold and 1.8-fold, respectively, whereas no significant increase in *XsGAS* expression was found in MeJA-treated plants. GA_3_ altered the *XsGAS* transcription in *X. strumarium* probably through a DELLAs/MYC2 complex that was previously discovered in *A. thaliana* plants [15]. Given that GA_3_ treatment did not affect the gland density/size (see the results described above), thus it might be inferred that the increse in xanthumin production by GA_3_ treatment was due to an increase in pathway gene expression within glandular trichomes. For the IAA treatment, it was hard to dissect whether the increase in *XsGAS* expression was attributable to the promoting effects of its treatment on gland cells. Transcript analysis on a single glandular trichome might be necessary to understand this. For the MeJA treatment, although it showed improved xanthium production (Figure 1) and gland cells (Figure 2), MeJA elicitation did not enhance the *XsGAS* expression (Figure 3A), suggesting that the increase in xanthumin production by MeJA elicitation was possibly, at least in part, due to its promoting effects on gland cells. In addition, since the production of xanthumin increased upon flowering initiation but was not accompanied by an increment in either gland density or gland size (Table 1), we examined whether the flowering initiation stimulated higher levels of *XsGAS* transcripts. As shown in Figure 3B, a 1-fold increase in *XsGAS* transcript levels was observed at the flowering initiation stage (100 days-old) relative to that of 80 days-old seedlings. On the basis of these results, we suggest that GA_3_ treatment or flowering initiation improved xanthumin production not by forming a higher gland density, but by up-regulating pathway gene expression within gland cells.

## 3. Experimental Section

### 3.1. Chemicals and Plant Materials

A xanthatin standard was purchased from the BioBioPha Company (Yunnan, China). Methyl jasmonate (MeJA) was from Sigma-Aldrich Company (Steinheim, Germany). Indol-yl-3-acetic acid (IAA) and gibberellic acid (GA_3_) were from Biosharp Company (Beijing, China). All the solvents used in this study were HPLC-grade. *X. strumarium* L*.* plants were from Nanyang County, Henan Province (China), and the species identification was confirmed by Jianqiang Li at the Wuhan Botanical Garden, Chinese Academy of Sciences (WBG-CAS). Its seeds were deposited in the Plant Library of WBG-CAS (ID no. is 2013718-10) to maintain the genetic homogeneity, and the seeds from one individual plant were used in this study.

### 3.2. Flowering Induction and Phytohormonal Elicitation

*X. strumarium* L*.* seeds were germinated and grown with 16 h light (150 µmol·m^−2^·S^−1^)/8 h dark cycles at 25 °C. For phytohormonal elicitations, 25 days-old seedlings with six expanded leaves per plant were chosen. MeJA, IAA, and GA_3_ solutions (dissolved in 0.01% ethanol) were sprayed in aliquots of 2.5 mL per plant at different concentrations. For MeJA or IAA treatment, concentrations of 50, 100, 200, and 400 µM were applied; for GA_3_ treatment, concentrations of 10, 20, 50, 100, 200, and 400 µM were used. Plants sprayed with 0.01% ethanol in water served as the mock controls. To minimize the errors possibly resulting from the differences between individual plants, 10 plants were sprayed for each concentration of the treatments. To prevent the emission of volatile phytohormones, the treated plants were covered by a clear plastic bag for 1 h allowing the elicitor solutions to be absorbed to a larger extent. After the treatments, the plants were further grown in 16 h light/8 h dark cycles at 25 °C for 7 days until the analysis. For inducing flowering, 80 days-old plants were transferred to 12 h light/12 h dark cycles at 25 °C. In total, 18 plants were used for the flowering induction. For the analysis, the first fully-expanded leaf from the top of the plants (the total area of the leaf was estimated to be about 8.0 cm^2^) was collected and cut into two halves along the leaf mid-vein, one half was used for xanthumin measurement and the other half was for the analysis of glandular trichomes.

### 3.3. Chemical Extraction and HPLC Analysis

HPLC analysis was performed using a Shimadzu LC-20AT HPLC system (Shimadzu, Kyoto, Japan) connected to a photodiode array detector. The column was an Inertsil ODS-SP reverse phase column (5 µm, 250 × 4.6 mm, Shimadzu). The wavelength was set from 190–800 nm. For determining xanthumin concentration, 300 mg of fresh material was grounded in liquid nitrogen and extracted with 3 mL of chloroform. The extracts were dried under reduced pressure and redissolved in 1.5 mL of methanol for HPLC analysis. The mobile phase, consisting of water (A) and acetonitrile (B) containing 0.1% (v/v) formic acid, was pumped at 0.8 mL/min in a step-wise gradient mode as follows: 0–20 min, 30% to 60% B; 20.1–30 min, 60% B; 30–30.1 min, 60%–30% B; 30.1–40 min, 30% B and the column oven temperature was 25 °C. A xanthumin standard is not commercially available so its production was roughly assessed by calculating the HPLC peak area of xanthumin. For analyzing GA_3_ concentration, 500 mg of the leave material was extracted overnight with 2.5 mL of methanol at 4 °C. The extract was centrifuged at 4000× *g* at 4 °C for 15 min, evaporated to dryness under vacuum at room temperature, and redissolved in 500 µL methanol. The methanol extract was then filtered through 0.45 µm inorganic membrane filter (Anpel, Shanghai, China) prior to HPLC analysis. The samples were eluted with water (A)-acetonitrile containing 0.1% formic acid (B) (A:B = 75:25, v/v) at a flow rate of 0.8 mL/min. The column oven temperature was set to 30 °C. The maximum wavelength for observing GA_3_ was set at 210 nm.

### 3.4. Glandular Trichome Analysis

A scanning electron microscopy (SEM, Quanta 150, Hillsboro, OR, USA) was used for the measurements of glandular trichome density and glandular trichome cross-sectional area. For measuring the gland density, the areas of leaf tip, leaf edge, and leaf center proximal to mid-vein [15], were chosen and the numbers of gland were counted in two 3 mm × 3 mm ocular grids per each area at 200× magnification under an accelerating voltage of 20 kv. For measuring gland size, the major (A) and minor (B) axes of the almost elliptical cross-sectional area of the gland were measured and the area was calculated according to the formula, area = ABπ/4 [18].

### 3.5. XsGAS Transcript Analysis

Consistent with the experiments above, the first leaf from the top of the plants was used for the RNA extractions. Plant RNA was extracted using the TRIzol reagent (Invitrogen, Carlsbad, CA, USA) according to the manufacturer’s protocol. First-strand cDNA was prepared from one microgram of the total RNA using SuperScript III reverse transcriptase (Invitrogen). The gene-specific primer sequences 5'-TCCGCCTTCTAACCCATGCGATAA-3' and 5'-GCAATGTCTTGGAACGCTTCTTTT-3' were used for real-time PCR analysis of *XsGAS* transcript. The length of the amplified *XsGAS* transcript is 179 bp. *X. strumarium* L*.* *Actin* gene (GenBank accession No. JF434698) was used as the reference gene to normalize the variations of the sample cDNA levels, and amplified with the specific primers 5'-TACTACAACGGCAGAACGGGAAA-3' and 5'-TCATAGACGGCTGGAACAAAACC-3'. Estimates of the gene transcripts were detected using the comparative threshold cycle method [21]. Real-time PCRs were performed on an ABI StepOne Plus Cycler (ABI, Forster, CA, USA) with FastStart Universal SYBR Green Master (ROX) (Roche, Mannheim, Germany). All the PCRs were carried out in two independent experiments with three technical repeats under the following conditions: 3 min at 94 °C; 30 s at 94 °C, 30 s at 58 °C, 30 s at 72 °C for 35 cycles, followed by the final extension at 72 °C for 7 min.

## 4. Conclusions

Flowering initiation and exogenous GA_3_ application were found to improve xanthumin production, but did not lead to a higher gland density on *X. strumarium* L. young leaves; on the other hand, for MeJA and IAA treatments, both applications at appropriate concentrations not only improved xanthumin production, but also increased the gland density/size; It was suggested that the increased glandular trichome number and gland size under MeJA and IAA treatments, at least in part, caused the increment in xanthumin accumulation, while increase in xanthumin production activated by flowering initiation or GA_3_ treatment was because of the increased expression of pathway genes within glandular trichomes.

## Supplementary Materials

Supplementary materials can be accessed at: http://www.mdpi.com/420-3049/19/9/12898/s1.

## Supplementary File

Supplementary File 1

## References

[1] Zhang X.Q., Ye W.C., Jiang R.W., Yin Z.Q., Zhao S.X., Mak T.C., Yao X.S. (2006). Two new eremophilanolides from *Xanthium sibiricum*. Nat. Prod. Res..

[2] Kim H.S., Lee T.S., Yeo S.W., Seong L.S., Yu T.S. (2003). Isolation and characterization of antitumor agents from *Xanthium strumarium* L.. Korean J. Biotechnol. Bioeng..

[3] Ramirez-Erosa I., Huang Y., Hickie R.A., Sutherland R.G., Barl B. (2007). Xanthatin and xanthinosin from the burs of *Xanthium strumarium* L. as potential anticancer agent. Can. J. Physiol. Pharmacol..

[4] Kim Y.S., Kim J.S., Park S.H., Choi S.U., Lee C.O., Kim S.K., Kim Y.K., Kim S.H., Ryu S.Y. (2003). Two cytotoxic sesquiterpene lactones from the leaves of *Xanthium strumarium* and their *in vitro* inhibitory activity on farnesyltransferase. Plant Med..

[5] Chen F.F., Hao F.H., Li C.F., Gou J.B., Lu D.Y., Gong F.J., Tang H.R., Zhang Y.S. (2013). Identifying three ecological chemotypes of *Xanthium strumarium* L. glandular trichomes using a combined NMR and LC-MS method. PLoS One.

[6] Yokotani-Tomita K., Kato J., Kosemura S., Yamamura S., Kushima M., Kakuta H., Hasegawa K. (1997). Light-induced auxin-inhibiting substance from sunflower seedlings. Phytochemistry.

[7] Raupp F.M., Spring O. (2013). New Sesquiterpene Lactones from Sunflower Root Exudate as Germination Stimulants for *Orobanche cumana*. J. Agric. Food Chem..

[8] Yang C., Yuan C., Jia Z. (2003). Xanthanolides, germacranolides, and other constituents from *Carpesium longifolium*. J. Nat. Prod..

[9] De Kraker J.W., Franssen M.C., de Groot A., Konig W.A., Bouwmeester H.J. (1998). (+)-Germacrene A biosynthesis. The committed step in the biosynthesis of bitter sesquiterpene lactones in chicory. Plant Physiol..

[10] Nguyen D.T., Gopfert J.C., Ikezawa N., Macnevin G., Kathiresan M., Conrad J., Spring O., Ro D.K. (2010). Biochemical conservation and evolution of germacrene A oxidase in asteraceae. J. Biol. Chem..

[11] Bertea C.M., Voster A., Verstappen F.W., Maffei M., Beekwilder J., Bouwmeester H.J. (2006). Isoprenoid biosynthesis in *Artemisia annua*: Cloning and heterologous expression of a germacrene A synthase from a glandular trichome cDNA library. Arch. Biochem. Biophys..

[12] Majdi M., Liu Q., Karimzadeh G., Malboobi M.A., Beekwilder J., Cankar K., Vos R., Todorovic S., Simonovic A., Bouwmeester H. (2011). Biosynthesis and localization of parthenolide in glandular trichomes of feverfew (*Tanacetum parthenium* L. Schulz Bip). Phytochemistry.

[13] Matsuo K., Ohtsuki K., Yoshikawa T., Shishido K., Yokotani-Tomita K., Shindo M. (2010). Total synthesis of xanthanolides. Tetrahedron.

[14] Hong G.J., Xue X.Y., Mao Y.B., Wang L.J., Chen X.Y. (2012). *Arabidopsis thaliana* MYC2 interacts with DELLA proteins in regulating sesquiterpene synthase gene expression. Plant Cell.

[15] Mannan A., Ahmed I., Arshad W., Hussain I., Mirza B. (2011). Effects of vegetative and flowering stages on the biosynthesis of artemisinin in Artemisia species. Arch. Pharm. Res..

[16] Richards D.E., King K.E., Ait-ali T., Harberd N.P. (2001). How gibberellin regulates plant growth and development: A molecular genetic analysis of gibberellin signaling. Annu. Rev. Plant Biol..

[17] Zhou Z.J., An L.J., Sun L.L., Zhu S.J., Xi W.Y., Broun P., Yu H., Gan Y.B. (2011). Zinc Finger Protein5 Is Required for the Control of Trichome Initiation by Acting Upstream of Zinc Finger Protein8 in *Arabidopsis thaliana*. Plant Physiol..

[18] Rowe H.C., Ro D.K., Rieseberg L.H. (2012). Response of sunflower (*Helianthus annuus* L.) leaf surface defenses to exogenous methyl jasmonate. PLoS One.

[19] Aerts R.J., Alarco A.M., de Luca V. (1992). IAAs induce tryptophan decarboxylase activity in radicles of catharanthus seedlings. Plant Physiol..

[20] Maes L., van Nieuwerburgh F.C., Zhang Y., Reed D.W., Pollier J., vande Casteele S.R., Inze D., Covello P.S., Deforce D.L., Goossens A. (2011). Dissection of the phytohormonal regulation of trichome formation and biosynthesis of the antimalarial compound artemisinin in *Artemisia annua* plants. New Phytol..

[21] Bovy A., de Vos R., Kemper M., Schijlen E., Almenar Pertejo M., Muir S., Collins G., Robinson S., Verhoeyen M., Hughes S. (2002). High-flavonol tomatoes resulting from the heterologous expression of the maize transcription factor genes LC and C1. Plant Cell.

