# Supplementary material for: GA3 and Other Signal Regulators (MeJA and IAA) Improve Xanthumin Biosynthesis in Different Manners in Xanthium strumarium L
Source: Molecules. 2014 Aug 25;19(9):12898–908. doi: 10.3390/molecules190912898 (PMC6271588; doi:10.3390/molecules190912898)
Supplement: Supplementary File 1 [file molecules-19-12898-s001.pdf]

Supplementary Material

Figure S1. The deduced amino acid alignment of XsGAS with several germacrene A synthases from other plant species. AaGAS, germacrene A synthase (AFK93531) from *Artemisia annua*; HaGAS1, germacrene A synthase (Q4U3F7) from *Helianthus annuus*; LsGAS1, germacrene A synthase (AAM11626) from *Lactuca sativa*.

```
HaGAS1    MAAVGASAT-PLTNTKSTAEPVRPVANFPPSVWGDLFLSFSLDKSIMEEYAEAMEEPKEQ
LsGAS1    MAAVEANGT-LQANTKTTTEPVRPLANFPPSVWGDRFLSFSLDNTELEGYAKAMEEPKEE
AaGAS     MAAVQANVTGIKENTKTSAEPVRPLANFPPSVWGDRFLSFSLDRSELERYAIAMEKPKED
XsGAS     MAAVGANAT-LLTNTKSTVEPVRPLANFPPSVWGDMFLSFSLDNSKMEEYAKAMEKPKQE

HaGAS1    VRRLILDPTMDSNKKLSLIYTVHRLGLTYMFLKEIEAQLDRLFKEFNLEDYVELDLYTIS
LsGAS1    VRRLIVDPTMDSNKKLSLIYSVHRLGLTYLFLQEIEAQLDNIFKAFKLQDYDEVDLYTTS
AaGAS     LRKLIVDPTMDSNEKLGLIYSVHRLGLTYMFLQEIESQLDKLFNKFSLQDYEEVDLYTIS
XsGAS     VRRLILDPTMDSNKKLSLIYVVHRLGLTYMFLKEIEGQLDRLFEEFNLEDYVDVDLHTIS

HaGAS1    INFQAFRHLGYKLPCDVFNKFKNDDSTTFKESITGDVRGMLGLYESAQLRLKGENILDEA
LsGAS1    INFQVFRHLGHKLPCDVFNKFKDSSSGTFKESITNDVKGMLGLYECAQLRLRGESILDEA
AaGAS     INFQVFRHLGYKLPCDVFNKFKDVSSGTFKASIMSDVRGMLGLYESAQLRIRGEKILDEA
XsGAS     INFQAFRHLGYKLPCDVFNKFKNNDSNAFKESIASDVRGLLGLYESAQLRVKGEKILDDA

HaGAS1    SAFAETKLKSLVNTLEGSLAQQVKQSLRRPFHQGMPMVEARLYFSNYQEECSAHDSILKL
LsGAS1    SAFTVTQLKSVVNTLEGKLAQQVLQSLKRPFHQGMPMVEARFYFSNYDEECSTHESLVKL
AaGAS     SVFTEAKLKSVVNTLEGDLAQQVTQSLRRPFHQGMPMVEARLYFSNYEKECSTYDSLLKL
XsGAS     SAFAETKLKSLVNTLEGSLAQQVKQALKRPFHQGMPMVEARLYFTNYQEEFSKYDSLLKL

HaGAS1    AKLHFNYLQLQQKEELRIVSQWWKDMRFQETTPYIRDRVPEIYLWILGLYFEPRYSLARI
LsGAS1    AKLHFNYLQLQQKEELRIVSKWWKDMRFQETTPYIRDRVPEIYLWILGLYFEPRYSLARI
AaGAS     AKLHFKYLELRQKEELRIVPKWWKDMRFHETTPYIRDRVPEIYLWILGLYFEPRYSLARI
XsGAS     AKLHFNYLQLQQKEELRIVSKWWKDMRFQETTPYIRDRVPEIYLWILGLYFEPKYSLARI

HaGAS1    IATKITLFLVVLDDTYDAYATIEEIRLLTDAINRWDISAMNQIPEYIRPFYKILLDEYAE
LsGAS1    IATKITLFLVVLDDTYDAYATIEEIRLLTDAINRWDISAIEQIPEYIRPFYKILLDEYAE
AaGAS     IATKITLFLVVLDDTYDAYATIEEIRPPTDAISKWDISAMEQIPEYIRPFYKILLDEYAE
XsGAS     IATKITLFLVVLDDTYDAYGTLEELRLLTHAINRWDMRAMSDIPEYIRPFYKILLDEYAE

HaGAS1    LEKQLAKEGRANSVIASKEAFQDIARGYLEEAEWTNSGYVASFPEYMKNGLITSAYNVIS
LsGAS1    LEKQLAKEGRAKSVIALKEAFQDIARGYLEEAEWTNSGYVASFPEYMKNGLVTSAYNVIS
AaGAS     IEKKMAKEGRANTVIASKEAFQDIARGYLEEAEWTNSGYVASFPEYMKNGLITSAYNVIS
XsGAS     LEKQLAKEGRLKSVIASKEAFQDIARGYIEEAEWTNSGYVASFPEYMKNGLITSAYNVIS

HaGAS1    KSALVGMGEIVSEDALVWYESHPQILQASELISRLQDDVMTYQFERERGQSATGVDSYIK
LsGAS1    KSALVGMGEMVSEDALAWYESHPKTLQASELISRLQDDVMTYQFERERGQSATGVDSYIK
AaGAS     KSALVGMGEIVSEDALAWYESHPKTLQASELISRLQDDVMTYQFERERGQSATGVDAYIK
XsGAS     KSALVGMGEVVSADALAWYESHPKILQASELISRLQDDVMTYQFERERGQSATGVDSYIK

HaGAS1    TYGVSEKVAIDELKKMIENAWKEINEGCLKPREVSMDLLAPILNLARMIDVVYRYDDGFT
LsGAS1    TYGVSEKEAIDELNKMIENAWKDINEGCLKPREVSMDLLAPILNLARMIDVVYRYDDGFT
AaGAS     TYGVSEKEAIDALKIMIENAWKDINEGCLKPRQVSMDLLAPILNLARMIDVVYRYDDGFT
XsGAS     TYGVSEKEAIEELKKMIENAWKDINEGCLKPREVSMDLLAPILNLARMIDVVYRYDDGFT

HaGAS1    FPGKTLKEYITLLFVGSSPM
LsGAS1    FPGKTMKEYITLLFVGSSPM
AaGAS     FPGKTLKEYINLLFVGSLPV
XsGAS     FPGKTLKEYITLLFVDSLPM
```
